# Supplementary material for: Bioactivity Screening and Gene-Trait Matching across Marine Sponge-Associated Bacteria
Source: Mar Drugs. 2021 Jan 30;19(2):75. doi: 10.3390/md19020075 (PMC7912018; doi:10.3390/md19020075)
Supplement: Supplementary file 1 [file marinedrugs-19-00075-s001.pdf]

Supplementary Material

Bioactivity Screening and Gene-Trait Matching Across Marine Sponge-Associated Bacteria

Asimenia Gavriilidou [1,*], Thomas Andrew Mackenzie [2], Pilar Sánchez [2], José R. Tormo [2], Colin Ingham [3], Hauke Smidt [1] and Detmer Sipkema [1]

[1] Laboratory of Microbiology, Wageningen University and Research, 6708 WE Wageningen, The Netherlands
[2] Fundación MEDINA, Centro de Excelencia en Investigación de Medicamentos Innovadores en Andalucía, Avda. del Conocimiento 34, 18016 Granada, Spain
[3] Hoekmine BV, Utrecht, The Netherlands
* Correspondence: asimenia.gavriilidou@wur.nl

Table of Contents

Figure S1 3

Table S1 4

Table S2 5

Table S3 8

Table S4 9

Table S5 11

References 12

Figure S1. KnownClusterBlast output of biosynthetic gene clusters (BGCs) predicted in strain Aa3_DN216_4B10_1 showing 100% homology to reference BGCs in the Minimum Information about a Biosynthetic Gene cluster (MIBiG) database [1]. Colors indicate BLAST matches of individual genes between query and reference sequences.

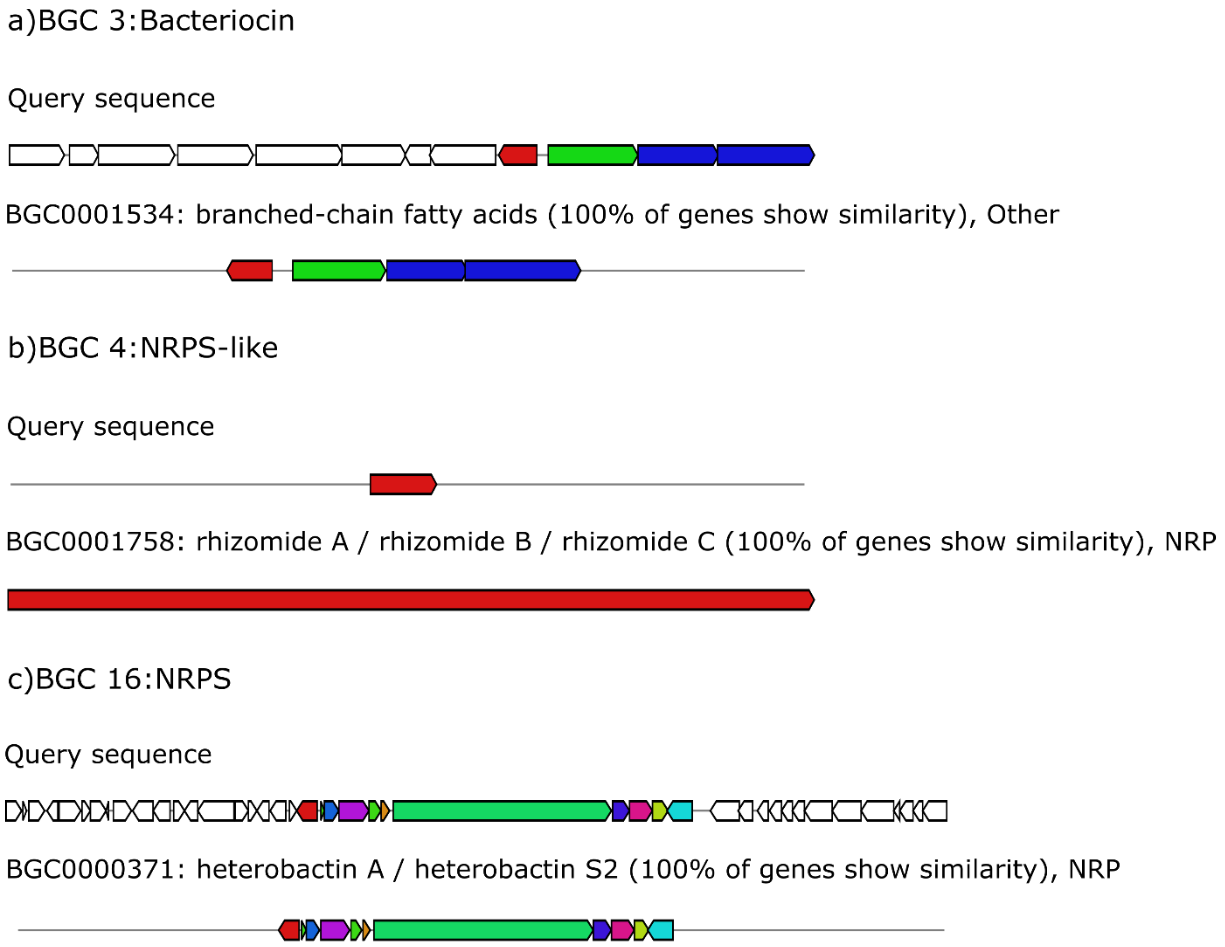

Table S1. Genome assembly metrics of strains included in the present study.

Strain ID	Isolation Source	Contigs	Genome size (Mb)	GC content (%)	N50 (Mb)	Completeness (%)	Contamination (%)	Coverage (X)	Total gene count
Aa3_DN55_6A7	*Aplysina aerophoba*	36	7.2	64.6	0.4	100.0	0.1	31	6655
Pf1_Ps_8H04_1	*Petrosia ficiformis*	18	5.7	48.2	0.5	100.0	0.2	295*	5174
Pf1_DN206_4B7	*Petrosia ficiformis*	52	5.1	52.8	0.4	100	0.8	226*	4692
Irc_Ps_AB108	*Ircinia* sp.	49	5.9	44.6	0.4	99.6	0.0	200*	5369
Pf1_DN64_8G1	*Petrosia ficiformis*	30	5.8	51.4	0.6	99.9	0.3	206	5275
Aa3_DN64_1D3	*Aplysina aerophoba*	22	5.8	50.3	0.5	99.6	0.3	711*	5239
Pf1_Ps_8H06	*Petrosia ficiformis*	40	6.1	49.7	0.4	99.6	0.6	264*	5554
Cn_Ps_AB111	*Chondrilla nucula*	36	5.9	49.8	0.4	99.9	0.0	228*	5423
Aa3_Str.68_7G12	*Aplysina aerophoba*	29	5.9	51.0	0.5	99.7	0.3	138*	5288
Acac_Ps_AB113	*Acanthella acuta*	13	5.4	51.0	0.9	99.6	0.0	191*	4886
Aa3_DN166_3E9_2	*Aplysina aerophoba*	40	4.6	56.2	0.2	99.1	0.0	237	4558
Pf1_DN81_6F7_2	*Petrosia ficiformis*	30	4.5	57.9	0.3	99.9	0.0	165	4356
Cc1_DN217_4H2	*Corticium candelabrum*	108	4.7	49.8	0.1	99.6	1.1	317	4229
Aa3_DN138_5C8	*Aplysina aerophoba*	46	3.3	41.4	0.2	100.0	0.0	310	3085
Pf1_DN14_7A9_1	*Petrosia ficiformis*	51	2.9	46.8	0.1	97.7	0.3	113	2434
Aa3_DN73_5E10_2	*Aplysina aerophoba*	41	2.6	47.0	0.1	92.2	0.3	385	2194
Aa3_DN30_1H2	*Aplysina aerophoba*	39	5.4	32.9	0.2	97.3	1.5	86	4684
Aa3_DN216_4B10_1	*Aplysina aerophoba*	79	7.1	62.5	0.3	99.9	1.1	43	6770
Aa3_DN213_3F7	*Aplysina aerophoba*	60	4.2	63.0	0.2	99.4	0.0	300	3850
Aa3_DN216_4B10_2	*Aplysina aerophoba*	19	3.4	72.9	0.3	99.8	0.0	51	3220
Aa3_DN71_7G3_2	*Aplysina aerophoba*	65	4.9	40.5	0.2	90.4	1.4	112	4807

*adapted from Versluis et al. [1]

Table S2. Taxonomic classification of studied strains based on 16S rRNA gene sequences (nr/nt database) and marker genes (GTDB database).

Strain ID	16S rRNA gene length (bp)	Best BLAST hit (accession number)	ID%	E-value	GTDB-Tk classification
Aa3_DN55_6A7	1340	*Bradyrhizobium* sp. LM6 (KX774628.1)	100.0	0.0	d__Bacteria;p__Proteobacteria;c__Alphaproteobacteria;o__Rhizobiales;f__Xanthobacteraceae;g__Bradyrhizobium;s__Bradyrhizobium sp003020075
Pf1_Ps_8H04_1	988	*Pseudovibrio* sp. MaPt6 (JX436420.1)	99.9	0.0	d__Bacteria;p__Proteobacteria;c__Alphaproteobacteria;o__Rhizobiales;f__Stappiaceae;g__Pseudovibrio;s__
Pf1_DN206_4B7	1337	*Pseudovibrio* sp. MA_AMC_33 (MN703974.1)	99.85	0.0	d__Bacteria;p__Proteobacteria;c__Alphaproteobacteria;o__Rhizobiales;f__Stappiaceae;g__Pseudovibrio;s__
Irc_Ps_AB108	1342	*Pseudovibrio* sp. 2011SOCNI15 (KF582860.1)	100.0	0.0	d__Bacteria;p__Proteobacteria;c__Alphaproteobacteria;o__Rhizobiales;f__Stappiaceae;g__Pseudovibrio;s__Pseudovibrio sp900143565
Pf1_DN64_8G1	1335	*Pseudovibrio* sp. 2011SOCNI15 (KF582860.1)	99.93	0.0	d__Bacteria;p__Proteobacteria;c__Alphaproteobacteria;o__Rhizobiales;f__Stappiaceae;g__Pseudovibrio;s__Pseudovibrio sp900143565
Aa3_DN64_1D3	1335	*Pseudovibrio* sp. 2011SOCNI15 (KF582860.1)	99.93	0.0	d__Bacteria;p__Proteobacteria;c__Alphaproteobacteria;o__Rhizobiales;f__Stappiaceae;g__Pseudovibrio;s__Pseudovibrio sp900143565

Pf1_Ps_8H06	1325	*Pseudovibrio* sp. 2011SOCNI42 (KF582882.1)	100.0	0.0	d__Bacteria;p__Proteobacteria;c__Alphaproteobacteria;o__Rhizobiales;f__Stappiaceae;g__Pseudovibrio;s__Pseudovibrio sp900143565
Cn_Ps_AB111	906	*Pseudovibrio* sp. 2011SOCNI15 (KF582860.1)	99.89	0.0	d__Bacteria;p__Proteobacteria;c__Alphaproteobacteria;o__Rhizobiales;f__Stappiaceae;g__Pseudovibrio;s__Pseudovibrio sp900143565
Aa3_Str.68_7G12	1325	*Pseudovibrio* sp. 2011SOCNI42 (KF582882.1)	100.0	0.0	d__Bacteria;p__Proteobacteria;c__Alphaproteobacteria;o__Rhizobiales;f__Stappiaceae;g__Pseudovibrio;s__Pseudovibrio sp900143565
Acac_Ps_AB113	1341	*Pseudovibrio* sp. ESS-18 (MH057247.1)	99.93	0.0	d__Bacteria;p__Proteobacteria;c__Alphaproteobacteria;o__Rhizobiales;f__Stappiaceae;g__Pseudovibrio;s__Pseudovibrio sp900143565
Aa3_DN166_3E9_2	1316	*Ruegeria* sp. 70077 (KX833139.1)	99.93	0.0	d__Bacteria;p__Proteobacteria;c__Alphaproteobacteria;o__Rhodobacterales;f__Rhodobacteraceae;g__Ruegeria;s__
Pf1_DN81_6F7_2	991	*Ruegeria atlantica* DN83_2B6 (KP769432.1)	100.0	0.0	d__Bacteria;p__Proteobacteria;c__Alphaproteobacteria;o__Rhodobacterales;f__Rhodobacteraceae;g__Ruegeria;s__
Cc1_DN217_4H2	1377	*Microbulbifer echini* ROA029 (MT510173.1)	99.85	0.0	d__Bacteria;p__Proteobacteria;c__Gammaproteobacteria;o__Pseudomonadales;f__Cellvibrionaceae;g__Microbulbifer;s__
Aa3_DN138_5C8	1396	*Acinetobacter radioresistens* OsEp_Plm_15B15 (MT367790.1)	100.0	0.0	d__Bacteria;p__Proteobacteria;c__Gammaproteobacteria;o__Pseudomonadales;f__Moraxellaceae;g__Acinetobacter;s__Acinetobacter radioresistens

Pf1_DN14_7A9_1	1373	*Psychrobacter celer* 7A3 (KU525106.1)	100.0	0.0	d__Bacteria;p__Proteobacteria;c__Gammaproteobacteria;o__Pseudomonadales;f__Moraxellaceae;g__Psychrobacter;s__Psychrobacter sp002810365
Aa3_DN73_5E10_2	1385	*Psychrobacter celer* G205M1 (MH256047.1)	100.0	0.0	d__Bacteria;p__Proteobacteria;c__Gammaproteobacteria;o__Pseudomonadales;f__Moraxellaceae;g__Psychrobacter;s__Psychrobacter sp002810365
Aa3_DN30_1H2	1044	*Aquimarina macrocephali* XH119 (KC178950.1)	100.0	0.0	d__Bacteria;p__Bacteroidota;c__Bacteroidia;o__Flavobacteriales;f__Flavobacteriaceae;g__Aquimarina;s__Aquimarina megaterium
Aa3_DN216_4B10_1	1068	*Rhodococcus erythropolis* KB1 (CP050124.1)	100.0	0.0	d__Bacteria;p__Actinobacteriota;c__Actinobacteria;o__Mycobacteriales;f__Mycobacteriaceae;g__Rhodococcus;s__Rhodococcus erythropolis_D
Aa3_DN213_3F7	1368	*Brevibacterium aurantiacum* SMQ-1419 (CP025333.1)	100.0	0.0	d__Bacteria;p__Actinobacteriota;c__Actinobacteria;o__Actinomycetales;f__Brevibacteriaceae;g__Brevibacterium;s__Brevibacterium aurantiacum
Aa3_DN216_4B10_2	1360	*Janibacter melonis* M0604 (KF924217.1)	99.93	0.0	d__Bacteria;p__Actinobacteriota;c__Actinobacteria;o__Actinomycetales;f__Dermatophilaceae;g__Janibacter;s__Janibacter melonis
Aa3_DN71_7G3_2	1397	*Bacillus frigoritolerans* ZB201705 (CP030063.1)	100.0	0.0	d__Bacteria;p__Firmicutes;c__Bacilli;o__Bacillales;f__Bacillaceae_A;g__Bacillus_X;s__Bacillus_X frigoritolerans

Table S3. Relative abundance of biosynthetic gene cluster (BGC) types identified by antiSMASH [2]. Numbers were normalized according to the genome size. NRPS; non-ribosomal peptide synthetase.

Strain ID	BGC types						Total BGCs
	Bacteriocin	NRPS	Terpene	Betalactone	Siderophore	Others	
Aa3_DN55_6A7	0.3	0.3	0.0	0.3	0.0	0.0	0.7
Pf1_Ps_8H04_1	0.2	0.0	0.2	0.2	0.0	0.4	0.9
Pf1_DN206_4B7	0.2	0.2	0.2	0.2	0.0	0.4	1.2
Irc_Ps_AB108	0.3	0.2	0.2	0.2	0.2	0.5	1.5
Pf1_DN64_8G1	0.3	0.2	0.2	0.3	0.2	0.7	1.9
Aa3_DN64_1D3	0.3	0.2	0.2	0.3	0.2	0.5	1.7
Pf1_Ps_8H06	0.2	0.2	0.2	0.2	0.0	0.7	1.3
Cn_Ps_AB111	0.3	0.0	0.2	0.2	0.0	0.3	1.0
Aa3_Str.68_7G12	0.2	0.2	0.2	0.2	0.2	0.7	1.5
Acac_Ps_AB113	0.4	0.0	0.2	0.2	0.2	0.4	1.3
Aa3_DN166_3E9_2	0.2	0.0	0.0	0.2	0.0	1.1	1.5
Pf1_DN81_6F7_2	0.2	0.0	0.0	0.2	0.0	0.9	1.3
Cc1_DN217_4H2	0.2	0.0	0.0	0.0	0.2	0.9	1.3
Aa3_DN138_5C8	0.0	0.0	0.0	0.6	0.3	0.3	1.2
Pf1_DN14_7A9_1	0.0	0.0	0.0	0.3	0.0	0.0	0.3
Aa3_DN73_5E10_2	0.0	0.0	0.0	0.4	0.0	0.0	0.4
Aa3_DN30_1H2	0.6	0.4	0.4	0.0	0.0	0.4	1.7
Aa3_DN216_4B10_1	0.1	1.0	0.1	0.0	0.0	1.5	2.8
Aa3_DN213_3F7	0.2	0.7	0.2	0.0	0.2	0.7	2.1
Aa3_DN216_4B10_2	0.0	0.0	0.3	0.0	0.3	0.6	1.2
Aa3_DN71_7G3_2	0.0	0.2	0.4	0.2	0.2	0.4	1.4

Table S4. Information on BGCs detected by antiSMASH [2] of strains selected for gene-trait matching and comparison to the Minimum Information about a Biosynthetic Gene cluster (MIBiG) database [3].

Strain ID	BGC	BGC type	Most similar known (MIBiG)	Similarity	MIBiG accession [3]
Aa3_DN216_4B10_1	1	NRPS, terpene	SF2575	6%	BGC0000269
	2	NRPS	coelichelin	27%	BGC0000325
	3	bacteriocin	branched-chain fatty acids	100%	BGC0001534
	4	NRPS-like	rhizomide A/rhizomide B/ rhizomide C	100%	BGC0001758
	5	NRPS	bacillomycin D	20%	BGC0001090
	6	NRPS	-	-	-
	7	NRPS	rifamorpholine A/rifamorpholine B/rifamorpholine C/rifamorpholine D/rifamorpholine E	4%	BGC0001759
	8	ectoine	ectoine	75%	BGC0000853
	9	NRPS	erythrochelin	57%	BGC0000349
	10	lanthipeptide	-	-	-
	11	PKS-like, amglyccycl	acarbose	7%	BGC0000691
	12	butyrolactone	-	-	-
	13	NRPS-like	-	-	-
	14	NRPS	chloramphenicol	17%	BGC0000893
	15	NRPS-like	thiotulin	8%	BGC0001193
	16	NRPS	heterobactin A/heterobactin S2	100%	BGC0000371
	17	LAP	diisonitrile antibiotic SF2768	11%	BGC0001574
	18	T1PKS	-	-	-
	19	T1PKS	kirromycin	8%	BGC0001070
	20	terpene	carotenoid	18%	BGC0000633
Pf1_DN64_8G1	1	acyl_amino_acids	-	-	-
	2	NRPS	rimosamide	14%	BGC0001760
	3	betalactone	pseudaminic acid	22%	BGC0001747
	4	T1PKS, T3PKS	-	-	-
	5	bacteriocin	-	-	-
	6	terpene	-	-	-
	7	betalactone	fengycin	13%	BGC0001095
	8	arylpolyene, ladderane	-	-	-

	9	siderophore	-	-	-
	10	bacteriocin	-	-	-
Aa3_DN64_1D3	1	betalactone	pseudaminic acid	22%	BGC0001747
	2	NRPS	rimosamide	14%	BGC0001760
	3	acyl_amino_acids	-	-	-
	4	arylpolyene, ladderane	-	-	-
	5	terpene	-	-	-
	6	bacteriocin	-	-	-
	7	siderophore	-	-	-
	8	bacteriocin	-	-	-
	9	betalactone	fengycin	13%	BGC0001095
	10	T3PKS, T1PKS	-	-	-
Aa3_DN213_3F7	1	NRPS	coelibactin	27%	BGC0000324
	2	NRPS	streptobactin	11%	BGC0000368
	3	NRPS-like	-	-	-
	4	siderophore	desferrioxamine E	50%	BGC0001478
	5	terpene	carotenoid	85%	BGC0000636
	6	LAP	corynazolicin	42%	BGC0001174
	7	ectoine	ectoine	75%	BGC0000853
	8	NRPS	caboxamycin	20%	BGC0001444
	9	bacteriocin	-	-	-

Table S5. Growth media and isolation source of the studied bacterial strains. Details on the media preparation can be found in the References mentioned below. MA; Marine Agar, MA/10; Marine Agar (10x diluted), MHA; Mueller-Hinton Agar, MHA/10; Mueller-Hinton Agar (10x diluted).

**Strain ID**	**Isolation source**	**Medium**	**Reference**
Aa3_DN55_6A7	*Aplysina aerophoba*	MA/10	[4]
Pf1_Ps_8H04_1	*Petrosia ficiformis*	MA	[1]
Pf1_DN206_4B7	*Petrosia ficiformis*	MA	[4]
Irc_Ps_AB108	*Ircinia* sp.	MA	[1]
Pf1_DN64_8G1	*Petrosia ficiformis*	MA	[4]
Aa3_DN64_1D3	*Aplysina aerophoba*	MA	[4]
Pf1_Ps_8H06	*Petrosia ficiformis*	MA	[1]
Cn_Ps_AB111	*Chondrilla nucula*	MA	[1]
Aa3_Str.68_7G12	*Aplysina aerophoba*	MA	[1]
Acac_Ps_AB113	*Acanthella acuta*	MA	[1]
Aa3_DN166_3E9_2	*Aplysina aerophoba*	MA	[4]
Pf1_DN81_6F7_2	*Petrosia ficiformis*	MHA	[4]
Cc1_DN217_4H2	*Corticium candelabrum*	MA	[4]
Aa3_DN138_5C8	*Aplysina aerophoba*	MA	[4]
Pf1_DN14_7A9_1	*Petrosia ficiformis*	MHA/10	[4]
Aa3_DN73_5E10_2	*Aplysina aerophoba*	MHA	[4]
Aa3_DN30_1H2	*Aplysina aerophoba*	MHA	[4]
Aa3_DN216_4B10_1	*Aplysina aerophoba*	MHA	[4]
Aa3_DN213_3F7	*Aplysina aerophoba*	MA	[4]
Aa3_DN216_4B10_2	*Aplysina aerophoba*	MHA	[4]
Aa3_DN71_7G3_2	*Aplysina aerophoba*	MHA	[4]

References

1. Versluis, D.; Nijsse, B.; Naim, M.A.; Koehorst, J.J.; Wiese, J.; Imhoff, J.F.; Schaap, P.J.; van Passel, M.W.J.; Smidt, H.; Sipkema, D. Comparative genomics highlights symbiotic capacities and high metabolic flexibility of the marine genus *Pseudovibrio*. *Genome Biol Evol* **2018**, *10*, 125-142.
2. Blin, K.; Shaw, S.; Steinke, K.; Villebro, R.; Ziemert, N.; Lee, S.Y.; Medema, M.H.; Weber, T. AntiSMASH 5.0: Updates to the secondary metabolite genome mining pipeline. *Nucleic Acids Res* **2019**, *47*, W81-W87.
3. Medema, M.H.; Kottmann, R.; Yilmaz, P.; Cummings, M.; Biggins, J.B.; Blin, K.; de Bruijn, I.; Chooi, Y.H.; Claesen, J.; Coates, R.C., *et al.* Minimum information about a biosynthetic gene cluster. *Nat Chem Biol* **2015**, *11*, 625-631.
4. Versluis, D.; McPherson, K.; van Passel, M.W.J.; Smidt, H.; Sipkema, D. Recovery of previously uncultured bacterial genera from three mediterranean sponges. *Mar Biotechnol* **2017**, *19*, 454-468.
